# Remission of Chimeric Antigen Receptor T-Cell-Refractory Diffuse Large B-Cell Lymphoma Resolved by Surgery and CD20 Bispecific Therapy

**DOI:** 10.7759/cureus.84617

**Published:** 2025-05-22

**Authors:** Vinny Lococo, Luke Selby, Rebecca Farmer, Janet Woodroof, Marc Hoffmann, Forat Lutfi

**Affiliations:** 1 Department of Hematologic Malignancies and Cellular Therapeutics, University of Kansas Medical Center, Kansas City, USA; 2 Department of Surgical Oncology, University of Kansas Medical Center, Kansas City, USA; 3 Department of Plastic, Wound, and Burn Surgery, University of Kansas Medical Center, Kansas City, USA; 4 Department of Pathology and Laboratory Medicine, University of Kansas Medical Center, Kansas City, USA

**Keywords:** cd20 biospecific t-cell engager therapy (btce), chimeric antigen receptor (car) t-cell therapy, diffuse large b cell lymphoma (dlbcl), dlbcl surgery, refractory dlbcl, surgical excision, tumor microenvironment (tme)

## Abstract

Diffuse large B-cell lymphoma (DLBCL) is the most common form of non-Hodgkin’s lymphoma. Some patients who progress after frontline chemoimmunotherapy can be cured with chimeric antigen receptor (CAR) T-cell therapy, though its success remains limited. While promising, the majority of patients relapse after CAR T-cell therapy, and there is no accepted standard of care. In the following case report, we present a patient with primary refractory DLBCL with an isolated bulky recurrence in his proximal thigh that did not respond to local radiation therapy, progressed after cluster of differentiation (CD)19 and CD22 directed autologous CAR T-cell therapy, and initially failed to respond to CD20 bispecific T-cell engager epcoritamab. He underwent complete surgical excision of the localized lesion followed by resumption of epcoritamab as maintenance therapy, leading to a durable remission. While surgery is not typically considered therapeutic for DLBCL, this case highlights the potential value of surgical debulking in DLBCL with an isolated recalcitrant lesion. We also discuss the potential influence of the tumor microenvironment and the use of surgery in complex cases like this.

## Introduction

Diffuse large B-cell lymphoma (DLBCL) is the most common subtype of non-Hodgkins lymphoma resulting from malignant B-cell proliferation [1]. It commonly presents with extra-nodal sites in the gastrointestinal tract and the skin [2]. Though new immunotherapies, such as chimeric antigen receptor (CAR) T-cell therapy and bispecific T-cell engager therapy (BTCE) targeted therapy, have shown promising success, many patients do end up with relapse and progression due to tumor evasion strategies such as antigen loss and changes in the tumor microenvironment [3-5]. Herein, we present a case of a patient experiencing treatment failure following two CAR T-cell therapies but responding to surgical excision of a sizable resistant mass while on maintenance immunotherapy.

## Case presentation

A 66-year-old male patient presented to our hospital for refractory stage III non-germinal center DLBCL. His initial care was performed at another institution, where he presented with a one-year progressive swelling of his left groin. He underwent a core biopsy of the left inguinal mass with pathology consistent with DLBCL, non-germinal center subtype. Initial staging positron emission tomography-computed tomography (PET-CT) showed hypermetabolic lymphadenopathy of the neck and pelvis with a 10 cm left inguinal mass. No extranodal disease was noted, making this a stage III disease by Ann Arbor staging. He was initiated on rituximab-cyclophosphamide, hydroxydaunorubicin, vincristine sulfate, and prednisone (R-CHOP) for six cycles. Interim PET-CT prior to cycle three showed partial response; however, at the end of treatment, PET-CT demonstrated persistent fludeoxyglucose (FDG)-avid left inguinal adenopathy. Biopsy of the left inguinal node showed a persistent cluster of differentiation (CD)5+ B-cell lymphoma. The patient underwent radiotherapy to the inguinal region with 5500 cGy given over 24 fractions with persistent FDG-avid adenopathy at the end of treatment PET-CT. Excisional biopsy revealed persistent DLBCL and he was then referred to our center for consideration of an immune effector cell (IEC) therapy given primary refractory disease.

The patient underwent CD19 CAR T-cell therapy with axicabtagene ciloleucel within two months of initial identification of primary refractory disease. His treatment course was complicated by grade 2 cytokine release syndrome (CRS) managed by three doses of tocilizumab. Initial day +30 PET-CT showed the patient was in complete metabolic remission. However, on day +103, he was found to have an isolated recurrence in his proximal left thigh that was separate from his previous surgical biopsy site. A core needle biopsy confirmed DLBCL with weakly CD22-positive staining. Given this, he was enrolled in a CD22 CAR T-cell therapy clinical trial with firicabtagene autoleucel. Polatuzumab vedotin and rituximab as well as rituximab, gemcitabine, and oxaliplatin (R-GemOx) were given as bridging with clinical progression to both regimens.

**Figure 1:**
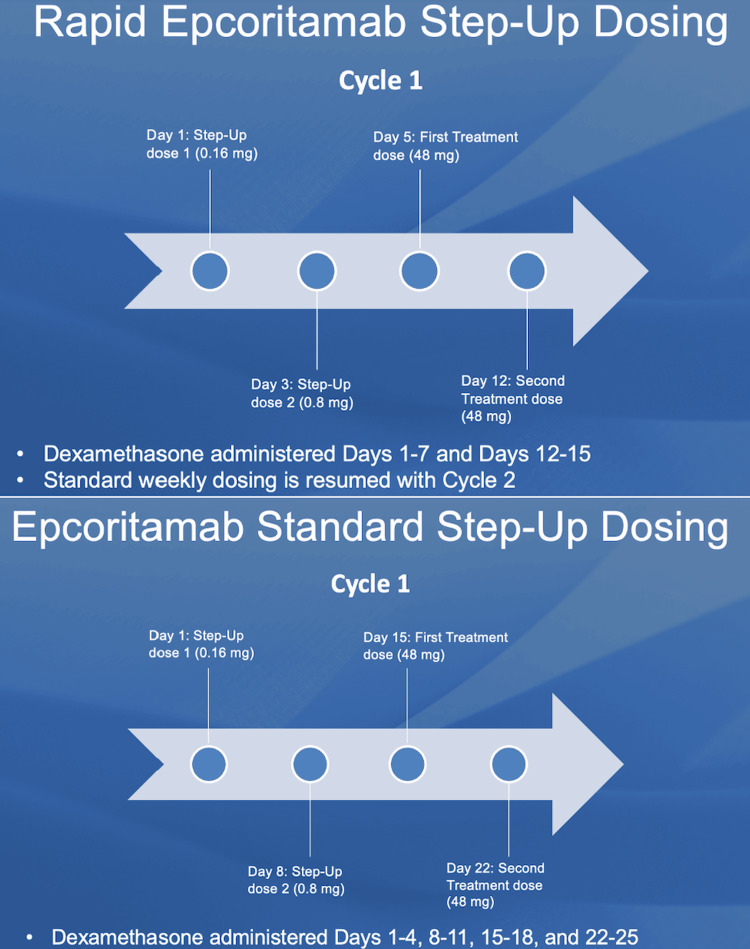
Epcoritamab standard versus rapid step-up dosing schedule.

The patient underwent an autologous CD22 CAR T-cell therapy infusion on trial. His treatment was complicated by grade 2 CRS, grade 1 immune effector cell-associated neurotoxicity syndrome (ICANS), neutropenic fevers, and acute respiratory failure with hypoxia during his hospital stay. On day +22, after receiving CD22 CAR-T, cells revealed the development of IEC-associated hemophagocytic lymphohistiocytosis (HLH) due to an uptrend in LDH (4,106 µ/L), ferritin (>30,000 ng/mL), and other features determined by an HScore summarized in Table 1. Day +30 restaging demonstrated a decrease in FDG avidity, but still significantly above liver background, consistent with a partial response by Lugano criteria. The patient’s left thigh mass continued to grow, tripling in size in a matter of months. Ultrasound biopsy revealed 50% DLBCL and 50% low-grade B-cell lymphoma consistent with relapsed disease. Both populations were CD20 positive and thus epcoritamab with rapid step-up was initiated (see Figure 1). The rapid step-up dosing was utilized to provide therapeutic benefit faster with the first three doses given in five versus the standard 15 days. He did not experience any grade CRS or ICANS with rapid step-up dosing.

**Table 1:** HScore Evaluation for Suspected hemophagocytic lymphohistiocytosis (HLH). *Defined as hemoglobin ≤9.2 g/dL, and/or white blood cell (WBC) ≤5,000/mm3 and/or platelets ≤110,000/mm3. **Hemoglobin, white blood cells, and platelets were 12.5 g/dL, 11,300/mm^3^, and 41,000/mm^3^ in this patient respectively. Note: HScore cutoff value of 169 corresponds to a sensitivity of 93%, specificity of 86%, and accurate classification of 90% of patients [6].

Parameter	Patient Value	H-Score Points	Reference Range
Known immunosuppression	Yes	18	Yes = 18 No = 0
Temperature, ˚C	36.7	0	<38.4 = 0, 38.4-39.4 = 33, >39.4 = 49
Organomegaly	No	0	No=0, Hepatomegaly or Splenomegaly=23, Hepatomegaly and Splenomegaly=38
Number of cytopenias*	1**	0	1 lineage=0, 2 lineages=24, 3 lineages=34
Ferritin, ng/mL	>30,000	50	<2,000 = 0, 2,000-6,000 = 35, >6,000 = 50
Triglycerides, mg/dL	314	44	<132.7 = 0, 132.7-354 = 44, >354 = 64
Fibrinogen, mg/dL	55	30	>250 = 0, ≤250 = 30
Aspartate Aminotransferase (AST), U/L	705	19	<30 = 0, ≥30 = 19
Hemophagocytic Features on Bone Marrow Aspirate	Yes	35	No = 0 Yes = 35
Total	-	196 (80-88% probably HLH)	0-337

**Figure 2:**
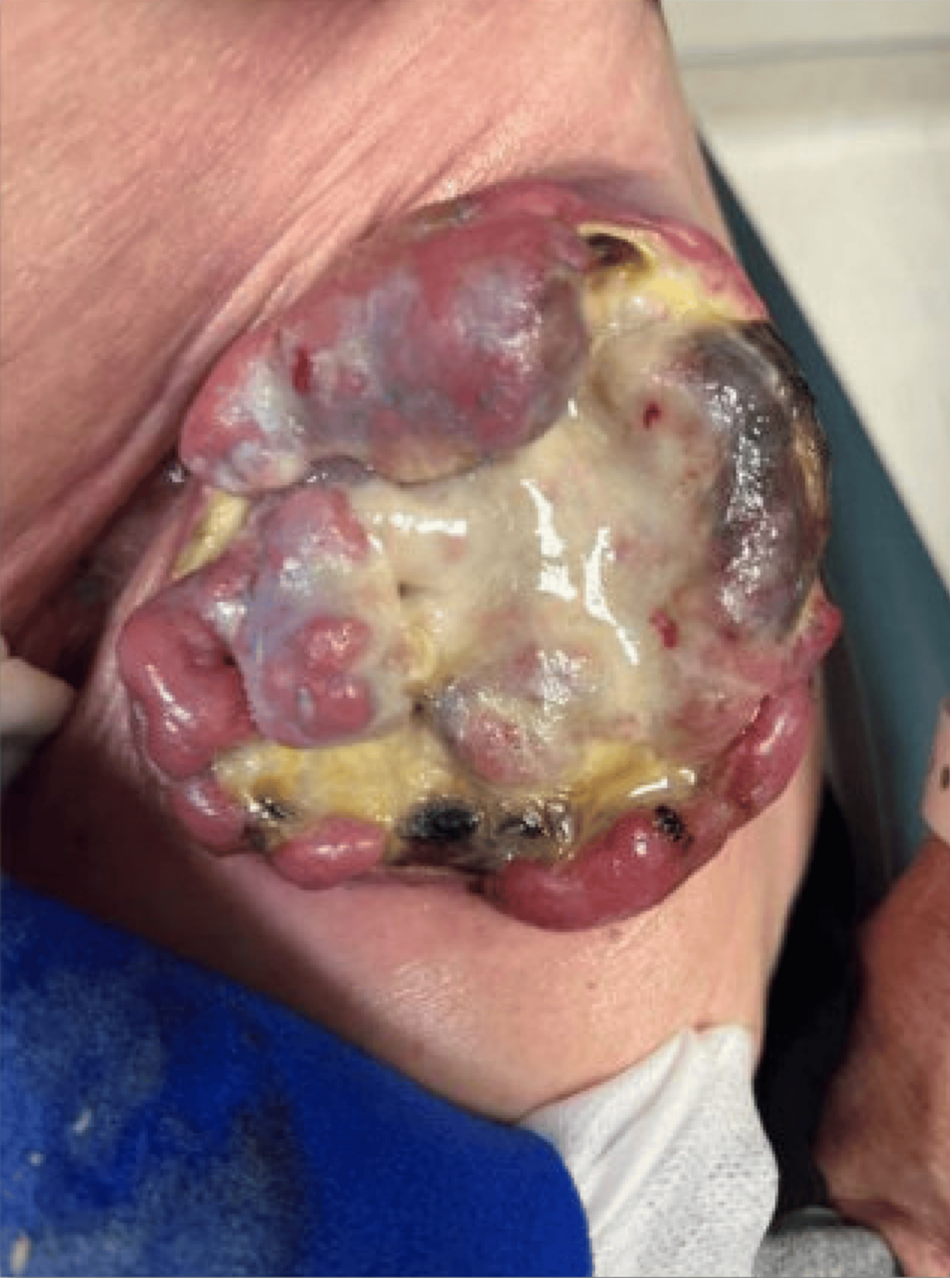
Left groin lesion 10x7cm prior to surgical resection.

**Figure 3:**
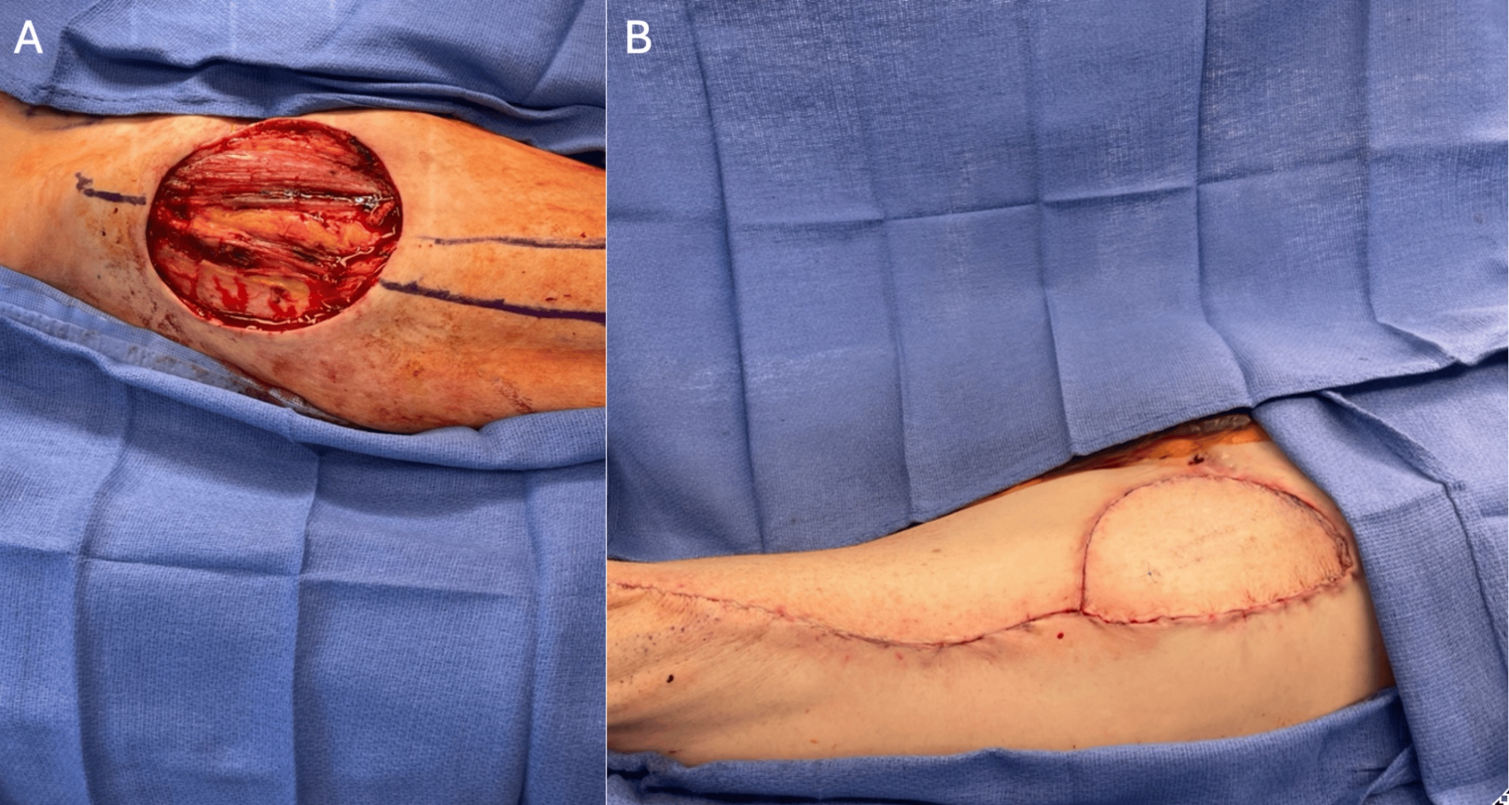
Intraoperative photos following surgical resection (A), followed by a fasciocutaneous flap (B).

**Figure 4:**
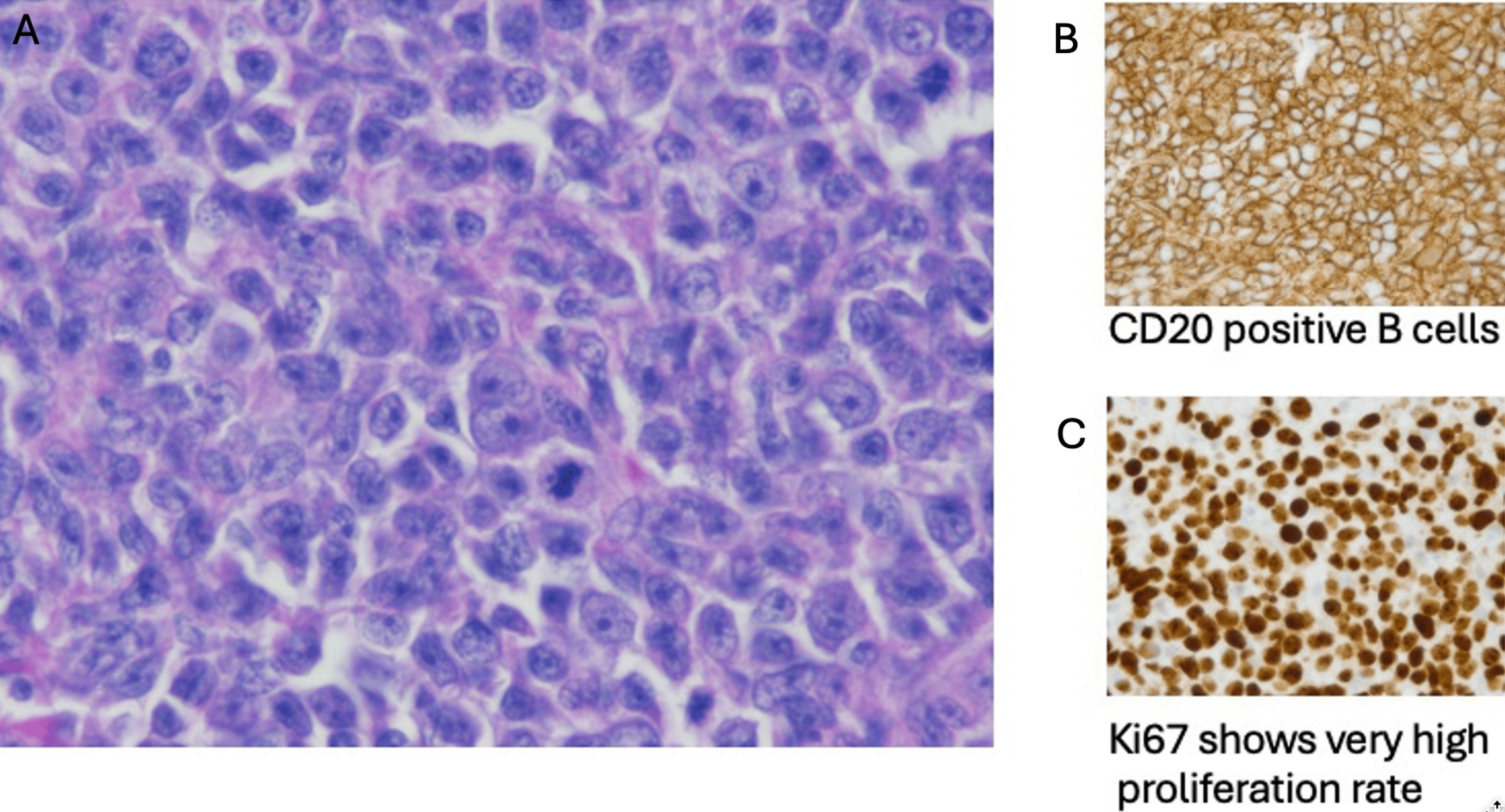
Histopathology of left thigh mass following resection demonstrating diffuse infiltrate of large atypical cells (A) along with CD20 staining (B) and Ki-67 stain (C) at 50x magnification.

**Figure 5:**
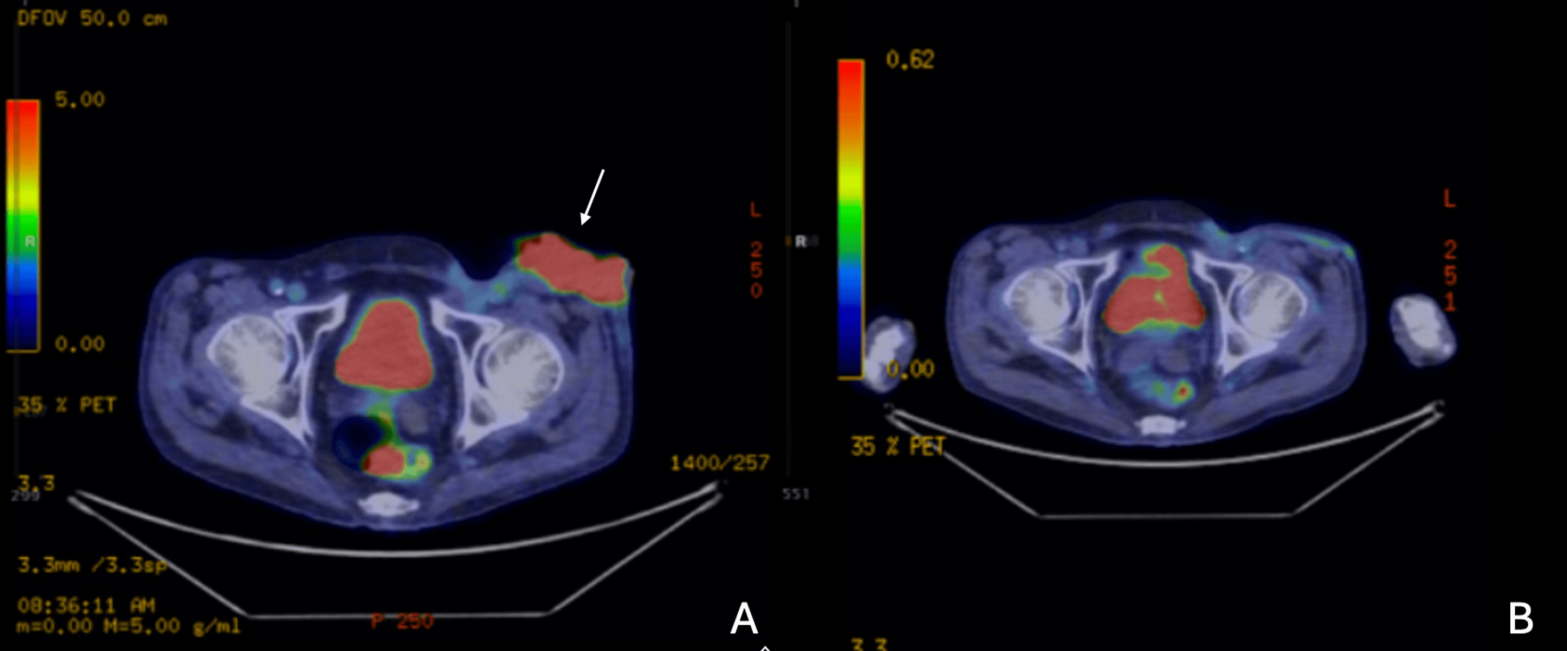
PET-CT imaging before resection (A) demonstrating left thigh mass (white arrow) and six weeks after surgical resection (B). Axial view PET/CT fused image. Image A demonstrates pre-resection of left posterior upper thigh soft tissue mass with SUV of 11.2 measuring 8x3cm, and image B demonstrates post-resection without evidence of lymphoma.

While on epcoritamab, the patient’s left thigh mass continued to grow to 10x7 cm (Figure 2). His restaging PET-CT after two cycles of epcoritamab confirmed local progression only without any evidence of distal disease. Thus, surgical oncology and plastic surgery were consulted for resection and immediate reconstruction. He underwent an uncomplicated radical resection of the proximal thigh mass with clinically negative margins. Tissue coverage required a fasciocutaneous flap, for which a pedicled anterolateral thigh flap was utilized (Figure 3). He had an uneventful post-operative recovery with no wound-healing complications. Histopathological analysis of the sample revealed the cells were morphologically similar to his previous biopsy (Figure 4). Interestingly, the tumor was still positive for CD19 and CD20 antigens by flow cytometry despite treatment with prior CD19 CAR-T and CD20 bispecific T-cell engager (BTCE) targeted therapies, possibly suggesting that resistance to therapy was achieved by tumor microenvironment rather than antigen loss. Restaging PET-CT six weeks after resection demonstrated a complete metabolic remission with a Deauville score of 1 (see Figure 5). Given ongoing CD20-positive disease and multiple relapses after chemoimmunotherapy, radiation therapy, two CAR-T therapies, BTCE therapy, and the patient’s explicit refusal to undergo a consolidative allogeneic hematopoietic stem cell transplant (alloHSCT) despite being a candidate, the patient was placed on epcoritamab maintenance. At eight months after surgical resection, the patient remains in complete remission with full function of his left lower extremity.

Written informed consent was obtained from the patient for publication of the case and images.

## Discussion

DLBCL is the most common lymphoid malignancy that arises in adulthood globally, accounting for about 31% of non-Hodgkin lymphoma in Western countries [7-9]. Though advancements in treatment regimens have improved, over 30% of patients will experience relapse and progression [7, 10]. Historically, treatment for refractory cases relies on systemic treatment and immunotherapy, such as CAR T-cell therapy. Surgery has not been known as a treatment of curative intent and is typically reserved for managing complications such as an obstruction from mass effect [11, 12]. However, recent emerging evidence suggests that surgery may provide a benefit in select cases. An earlier retrospective study done by Romagurea et al. at the M.D. Anderson Cancer Center revealed improved seven-year survival rates following surgical debulking for patients with stage I-II DLBCL compared to patients who did not undergo debulking (93% vs 35%) [13]. Wilder et al. confirmed these results through a case-control study of stage I-II aggressive lymphoma patients treated with CHOP and radiation therapy [14]. At five years, patients undergoing debulking surgery had improved overall survival (83% vs 71%) and progression-free survival (88% vs 62%) compared to patients not undergoing surgery [14]. More recently, Schmitz et al. evaluated the impact of complete surgical resection in aggressive non-Hodgkin lymphoma treated with immunotherapy [15]. The data found that patients with DLBCL treated with complete surgical resection were found to have a higher complete remission rate compared to those who were having an incomplete resection (100% vs 82.4%) [15]. Furthermore, the two-year overall survival (100% vs 95.9%) and progression-free survival (100% vs 92.2%) were improved for patients younger than 60 years [15]. Though most of the existing literature draws on patients with early-stage DLBCL, the principles from those studies may extend to select cases of localized relapse in advanced-stage patients. This aligns with our case, where surgical excision of a refractory mass followed by CD20 BTCE maintenance led to remission in a patient after failing two lines of CAR T-cell therapy. These findings suggest that when combined with immunotherapy, surgery may have a synergistic benefit in select patients with localized relapse.

Herein, we present a unique case where a patient with DLBCL refractory to multiple therapies, including CD19 and CD22-directed CAR-T cell therapies. It was not until a surgical resection of his large left thigh mass, followed by CD20 BTCE maintenance, that he achieved a durable remission. This is an unusual response to therapy, which raises questions regarding the mechanism of resistance. We propose that the tumor microenvironment played a significant role in resistance to treatment. This is evident as the distant disease sites responded to treatment, unlike the mass in the left thigh. Additionally, this mass demonstrated retained expression of CD19 and CD20, making antigen loss an unlikely resistance mechanism.

These findings emphasize the complexity of DLBCL, even in the era of immunotherapies. While CAR T-cell therapy has revolutionized the treatment of hematological malignancies, localized resistance can develop and negatively impact patient outcomes, thus signifying the potential need for other treatment options. Within the literature and the case we have presented, we demonstrate that surgery may be a suitable option for DLBCL. It is still under debate why surgery works in these studies, with many hypotheses being offered. One hypothesis points to the role of the tumor's microenvironment, which can “defend” malignant cells from the effects of cytotoxic chemotherapy and immunotherapy [5,14]. Further investigation is needed to determine who is a candidate and when surgery should be integrated as part of a patient’s treatment plan.

## Conclusions

In this report, we present a case of a patient with DLBCL who was refractory to multiple lines of systemic therapy including radiation therapy and CD19 and CD22 CAR T-cell therapies with a persistent and enlarging soft tissue mass of his thigh who achieved durable remission through a multifactorial strategy including surgical resection of the thigh mass and maintenance epcoritamab. Resection of isolated lesions that can render a patient disease-free with minimal surgical morbidity should be considered in highly selected cases of DLBCL that have not responded to systemic therapy.
